# NR6A1 regulates lipid metabolism through mammalian target of rapamycin complex 1 in HepG2 cells

**DOI:** 10.1186/s12964-019-0389-4

**Published:** 2019-07-17

**Authors:** Yinfang Wang, Xiaohong Wan, Yilong Hao, Yuanyuan Zhao, Lanlan Du, Yitong Huang, Zongjun Liu, Ying Wang, Nanping Wang, Peng Zhang

**Affiliations:** 1 Shanghai Putuo Central School of Clinical Medicine, Anhui Medical University, Hefei, 230001 China; 2 Central Laboratory, Putuo Hospital, Shanghai University of Traditional Chinese Medicine, Shanghai, 200062 China; 3 Department of Cardiovascular Medicine, Putuo Hospital, Shanghai University of Traditional Chinese Medicine, Shanghai, 200062 China; 4 Department of Biochemistry and Molecular Biology, Mayo Clinic, Florida, 32224 USA; 5 The Advanced Institute for Medical Sciences, Dalian Medical University, Dalian, 116044 China

**Keywords:** Lipogenesis, HepG2 cells, NR6A1, Insulin receptor, miR-205-5p

## Abstract

**Background:**

Lipogenesis is required for the optimal growth of many types of cancer cells, it is shown to control the biosynthesis of the lipid bilayer membrane during rapid proliferation and metastasis, provides cancer cells with signaling lipid molecules to support cancer development and make cancer cells more resistant to oxidative stress-induced cell death. Though multiple lipogenic enzymes have been identified to mediate this metabolic change, how the expression of these lipogenic enzymes are transcriptionally regulated remains unclear.

**Methods:**

Gain- and loss-of-function experiments were conducted to assess the role of transcriptional repressor, nuclear receptor sub-family 6, group A, member 1 (NR6A1) in HepG2 cells. RT-qPCR method was performed to investigate target gene of NR6A1. Western blot was employed to determine the mechanisms by which NR6A1 regulates lipid accumulation in HepG2 cells.

**Results:**

We provide evidence that NR6A1 is a novel regulator of lipid metabolism in HepG2 cells. NR6A1 knockdown can increase lipid accumulation as well as insulin-induced proliferation and migration of HepG2 cells. The lipogenic effect correlated well with the expression of lipogenic genes, including fatty acid synthase (FAS), diglyceride acyltransferase-2 (DGAT2), malic enzyme 1 (ME1), microsomal triglyceride transfer protein (MTTP) and phosphoenolpyruvate carboxykinase (PEPCK). NR6A1 knockdown also increased the expression of carnitine palmitoyltransferase 1A (CPT1a), the rate-limiting enzyme in fatty acid oxidation. Furthermore, NR6A1 knockdown induced lipid accumulation through mammalian target of rapamycin complex 1 (mTORC1), but not mTORC2. Moreover, siRNA-mediated knockdown of NR6A1 increased expression of insulin receptor (INSR) and potentitated insulin-induced phosphorylation of mTOR and AKT partly via miR-205-5p in HepG2 cells.

**Conclusions:**

These findings provide important new insights into the role of NR6A1 in the lipogenesis in HepG2 cells.

**Graphical abstract:**

.
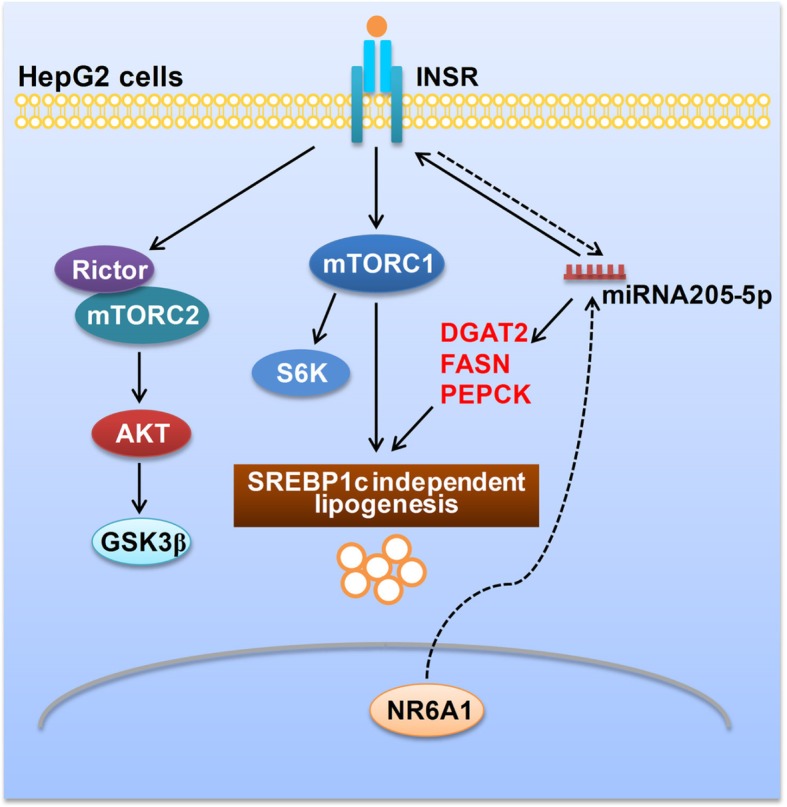

**Electronic supplementary material:**

The online version of this article (10.1186/s12964-019-0389-4) contains supplementary material, which is available to authorized users.

## Background

Many types of cancer cells exhibit increased capacity of exogenous lipid uptake and lipogenesis. This aberrant lipid metabolism contributes to the cell membrane synthesis, cell growth and transformed phenotype of cancer cells [1]. Diabetes mellitus, which is associated with hyperinsulinemia, is associated with an increased risk for development of liver cancer. Liver cancer and insulin resistance share a number of metabolic risk factors including insulin resistance and hyperinsulinism, which have been shown to result in abnormal cancer cell differentiation, proliferation and carcinogenesis. Insulin has been long assumed as a biological connection between metabolic disorders and cancers. Specifically, insulin was shown to promote carcinogenesis in vitro and in vivo [2, 3]. Activation of insulin receptor (INSR) in cancer cells triggers the signaling cascades of phosphatidylinositol 3-kinase (PI3K)-AKT pathway, which promotes proliferation and glycolysis but inhibits apoptosis [4]. Moreover, insulin leads to lipid metabolic reprogramming, a hallmark of cancer [5, 6]. Furthermore, insulin can activate the expression of lipid biosynthesis genes in cancer cells through stimulating AKT downstream effector, mammalian target of rapamycin complex 1 (mTORC1). But the molecular mechanism responsible for the increased risk of liver cancer in diabetes patients is yet not completely understood.

The nuclear receptor sub-family 6, group A, member 1 (NR6A1), which is also known as germ cell nuclear factor, is a member of the nuclear receptor superfamily of ligand-activated transcription factors. The NR6A1 knockout animal is embryonic lethal due to cardiovascular defects and failure to establish the correct chorioallantoic connection [7, 8]. Conditional ablation of NR6A1 revealed its important function in reproduction [9]. NR6A1 can bind to DNA sequences consist of a directly repeated (DR0) consensus motif PuGGTCA with zero spacing and acts as a transcriptional repressor. It has been reported that NR6A1 regulates differentiation of stem cells by suppressing pluripotency genes like Nanog and Oct4 [10]. It also binds to the DR0 element of promoters of teratocarcinoma-derived growth factor 1, bone morphogenetic protein-15 and growth differentiation factor-9 in vitro [11, 12]. Meanwhile, NR6A1 also regulates transcription in an indirect way by competing with transcription activator and regulating miRNAs. For example, NR6A1 competes with cAMP responsive element modulator τ for the same binding site in the promoter of mitochondrial glycerol-3-phosphate dehydrogenase [13, 14]. In vascular smooth muscle cells, NR6A1 modulates secreted phosphoprotein 1 expression via its binding with cAMP response element-binding protein [15]. Recently, it is revealed that NR6A1 controls expression of cyclin D1 via inhibition of miR-302a [16].

In this study, we demonstrated that NR6A1 regulates lipogenic gene expression and lipid content in HepG2 cells in an mTORC1-dependent manner. We further showed that NR6A1 suppresses miR-205-5p expression to indirectly induce INSR expression in HepG2 cells.

## Materials and methods

### Cell culture and reagents

The HepG2 hepatoma and HEK 293 cell lines obtained from American Type Culture Collection (Manassas, VA, USA) were maintained routinely in Dulbecco’s Modified Eagle’s Medium (DMEM) containing 10% fetal calf serum (FBS), 1% L-glutamine, 100 U/ml penicillin and 100 U/ml streptomycin under 5% CO2 at 37 °C. Opti-MEM and Lipofectamine RNAi reagents were purchased from Invitrogen (Carlsbad, CA, USA). RNAiso Plus was obtained from Clontech (Mountain View, CA, USA). Recombinant human insulin was purchased from R&D Systems (Minneapolis, MN, USA). Oleic acid (OA), palmate (PA) and Oil red O were obtained from Sigma-Aldrich (Munich, Germany). Rapamycin was purchased from Selleck company (Houston, TX, USA). Antibodies against NR6A1 and horseradish peroxidase–conjugated goat anti-rabbit or anti-mouse secondary antibodies were purchased from Santa Cruz Biotechnology (Dallas, TX, USA). Antibodies against phospho-Akt (Ser473), Akt, phospho-mTOR (Ser2448), mTOR, phospho-Glycogen synthase kinase 3 (GSK3β) (Thr389), phospho-S6kinase (S6K) (Ser371), S6K, phospho-INSR (Tyr1150/1151) and INSR were obtained from Cell Signaling Technology (Danvers, MA, USA).

### Animals

All the animal experiments in this study were conducted following the Guide for the Care and Use of Laboratory Animals published by the US National Institute of Health, 8th Edition (2011) and approved by the Institutional Animal Care and Use Committee of Putuo Hospital, Shanghai University of Traditional Chinese Medicine. Briefly, C57BL/6, Lepr^db/db^ male mice, Wistar and Goto-Kakizaki (GK) rats were housed with free access to food and water on a 12 h light/dark cycle. Blood samples were collected from the tail for examination of fasting blood glucose levels. The animals with elevated blood glucose had food with drawn for 16 h, with ad libitum access to water, prior to sacrifice. Liver tissue was collected to measure the NR6A1 expression.

### Small-interfering RNAs (siRNAs) and recombinant adenovirus

siRNAs targeting human NR6A1, Rictor, PEPCKc, SREBP1c and INSR were synthesized and transfected into cells by using LipofectamineRNAi reagent according to the manufacturer’s protocol. Transfection with scrambled siRNA was used as the control. The siRNA sequences are shown in Additional file 1: Table S1. The adenovirus expressing NR6A1 was expanded and purified as we previously reported [15].

### Total RNA extraction and quantitative PCR (qPCR)

Total RNA was isolated by using RNAiso Plus reagent. cDNA was synthesized with 1 μg of total RNA by using SuperScript II Reverse Transcriptase (Thermo Fisher Scientific, Waltham, MA, USA). The qPCR was performed with SYBR Green Master Mix (Thermo Fisher Scientific). Primer sequences were listed in the Additional file 1: Table S1.

### miRNA transfection and expression

The biological targets of miRNA were predicted by miRBase online database. The miR-205-5p duplexes were chemically synthesized. HepG2 transfection was performed with miRNA at a final concentration of 50 nM by using LipofectamineRNAi reagent. The miRNAs were extracted from samples containing total RNA with the miRNeasy mini kit (Qiagen, Hilden, Germany) following manufacturer’s instruction. Single-stranded cDNA was synthesized by miRcute Plus miRNA First-Strand cDNA Kit (TIANGEN Biotech, Beijing, China) from 1 μg of total RNA in a 15 μl reaction. The reactions were incubated sequentially at 42 °C for 60 min and then at 95 °C for 3 min. The qPCR was performed with SYBR Green Master Mix. The miRNA primer sequences were listed in the Additional file 1: Table S1.

### Western blot analysis

HepG2 cells were harvested and homogenized in ice cold RIPA lysis buffer (Thermo Fisher Scientific, Waltham, MA, USA) in the presence of proteinase inhibitor cocktail. Equal amounts of total proteins were resolved on SDS-polyacrylamide gels and then transferred onto polyvinylidene difluoride membranes (Amersham Biosciences). The membranes were blocked with blocking buffer (Tris-buffered saline, 5% bovine serum albumin, 0.1% Tween 20) at room temperature for 1 h. The target proteins were probed with primary antibodies at 4 °C overnight followed by the corresponding secondary antibodies conjugated with horseradish peroxidase for 1 h at room temperature. Protein bands on the membranes were visualized with Enhanced Chemiluminescence Plus (GE Life Sciences, Little Chalfont, United Kingdom).

### Oil red O staining

To examine the cellular neutral lipid droplet accumulation, HepG2 cells were washed with PBS and fixed in 4% paraformaldehyde for 15 min at room temperature. After fixation, cells were washed and stained with the working solution of Oil Red O in 60% isopropanol for 30 min at room temperature. After staining, the HepG2 cells were washed with water to remove unbound dye and examined by using a light microscope. To quantify Oil Red O content levels, the samples were dissolved with 100% isopropanol at room temperature for 5 min. The absorbance was read at 510 nm with a spectrophotometer.

### Reporter constructs and dual luciferase assay

The pmirGLO Dual-Luciferase miRNA Target Expression Vector (Promega, CA, USA) containing cDNA sequences encoding the Firefly luciferase reporter gene and the Renilla luciferase gene was linearized with Xho1 restriction enzyme. Fragments of 3′ INSR UTR containing one putative miR-205-5p seed match site (525–531 nt of 3′ INSR UTR) was PCR-amplified and cloned into the pmirGLO vector by using In-Fusion Dry-Down PCR Cloning Kits. Plasmids were purified using QIAGEN Plasmid Midi Kits. Transfection was performed with Lipofectamine 2000 reagent (Invitrogen). At 24 h post-transfection, firefly and renilla luciferase activities were measured by using Dual-luciferase Reporter Assay System (Promega, Madison, WI, USA) according the manufacturer’s protocol.

### Cell proliferation and migration assay

Cell proliferation was measured by Cell Counting Kit-8 (CCK-8) assay according to the manufacturer’s instruction (Sigma-Aldrich, Munich, Germany). For migration assay, confluent cells were made quiescent for 8 h in medium containing 0.5% FBS. The physical scratch wound was made with a sterile pipette tip. Detached cells were washed away with PBS. The cells were further cultured in fresh culture medium containing insulin (10 nM) for 24 h and then the healing of the scratch wound was monitored under phase-contrast microscopy and photographed. The rate of migration was measured by quantifying the area moved from the edge of the scratch toward the center of the scratch. The scratch wound closure was analyzed with Image J software.

### Statistical analysis

The control group was normalized to 1 in each single experiment. All the values were expressed as mean ± SEM. Statistical differences were determined to be significant at *P* < 0.05. Statistical significance was calculated by one-way ANOVA and Tukey multiple comparison test. All the experiments were routinely repeated at least 3 times.

## Results

### NR6A1 regulates insulin-stimulated proliferation and migration of HepG2 cells

**Fig. 1:**
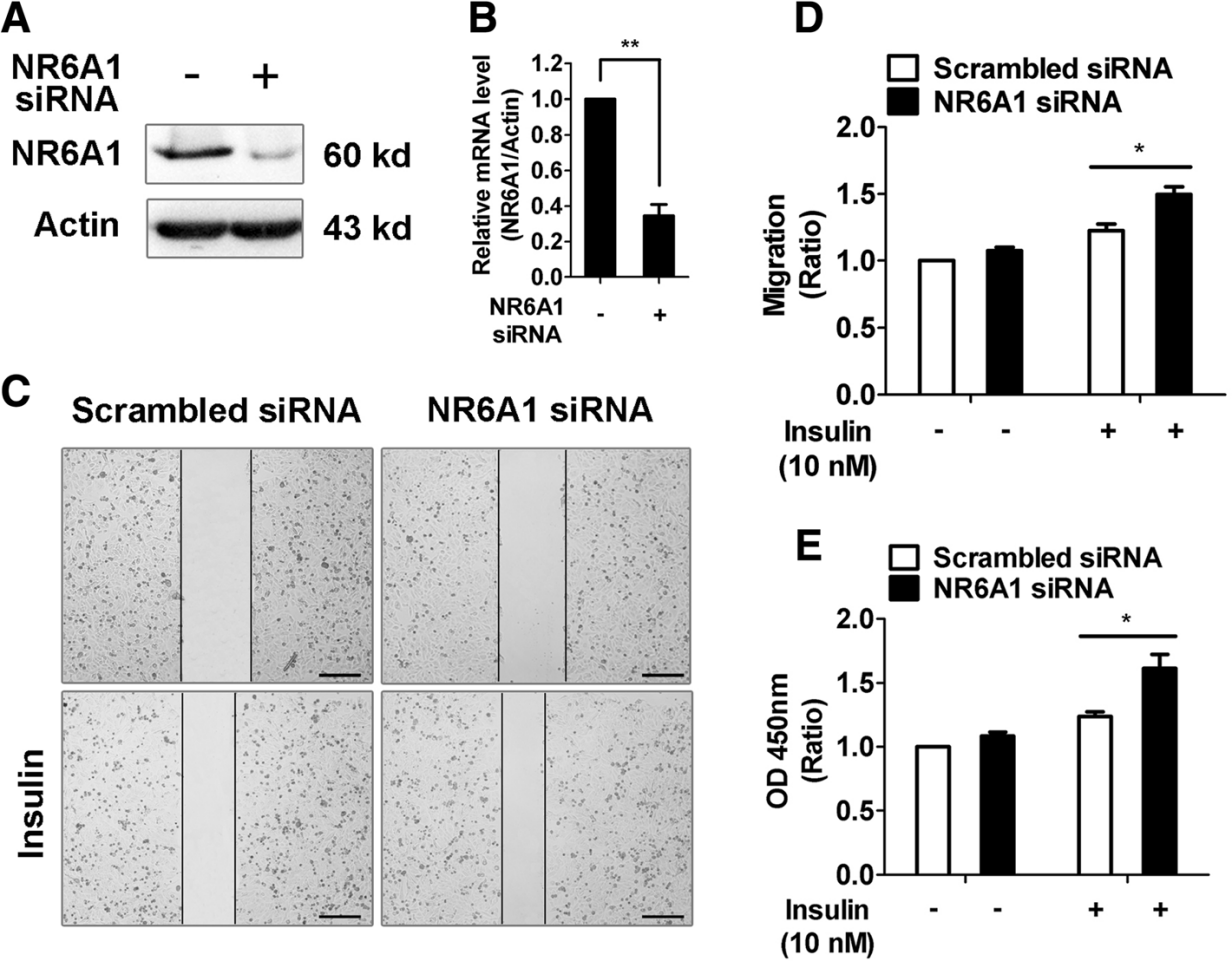
The effect of NR6A1 knockdown on insulin-induced proliferation and migration in HepG2 cells. HepG2 cells were transfected with scrambled or NR6A1 siRNA for 48 h. Western blotting (**a**) and qPCR assay (**b**) were used to confirm NR6A1 knockdown efficiency. Representative blots were shown. *n* = 3. (**c**) The insulin (10 nM) stimulated migration of control and NR6A1 knockdown cells was analyzed by a typical scratch wound healing assay. Representative images were shown. Bar = 200 μm. n = 3. (**d**) The ratio of wound closure compared with control. n = 3. *P < 0.05. (E) Control and NR6A1 knockdown HepG2 cells were treated with vehicle or insulin (10 nM) for 24 h. Cell proliferation was measured using CCK-8 proliferation kit. Results were obtained from 3 independent experiments. *P < 0.05

To study the effects of NR6A1 on insulin-induced proliferation and migration, a widely used hepatocellular carcinoma cell line, HepG2 cells were cultured and transfected with NR6A1 siRNA. Western blot and qPCR analysis confirmed that the protein and mRNA expression of NR6A1 was markedly reduced (Fig. 1A, B). Our results showd that NR6A1 knockdown slightly increased the migration and proferliation of HepG2 at basal level, consistent with previous studies [17, 18], administration of insulin induces greater migration and proliferaton of HepG2 cells. Importantly, NR6A1 knockdown significantly enhanced insulin-induced migration as well as proliferation in HepG2 cells (Fig. 1C-E).

### NR6A1 silencing increases lipid content in HepG2 cells

**Fig. 2:**
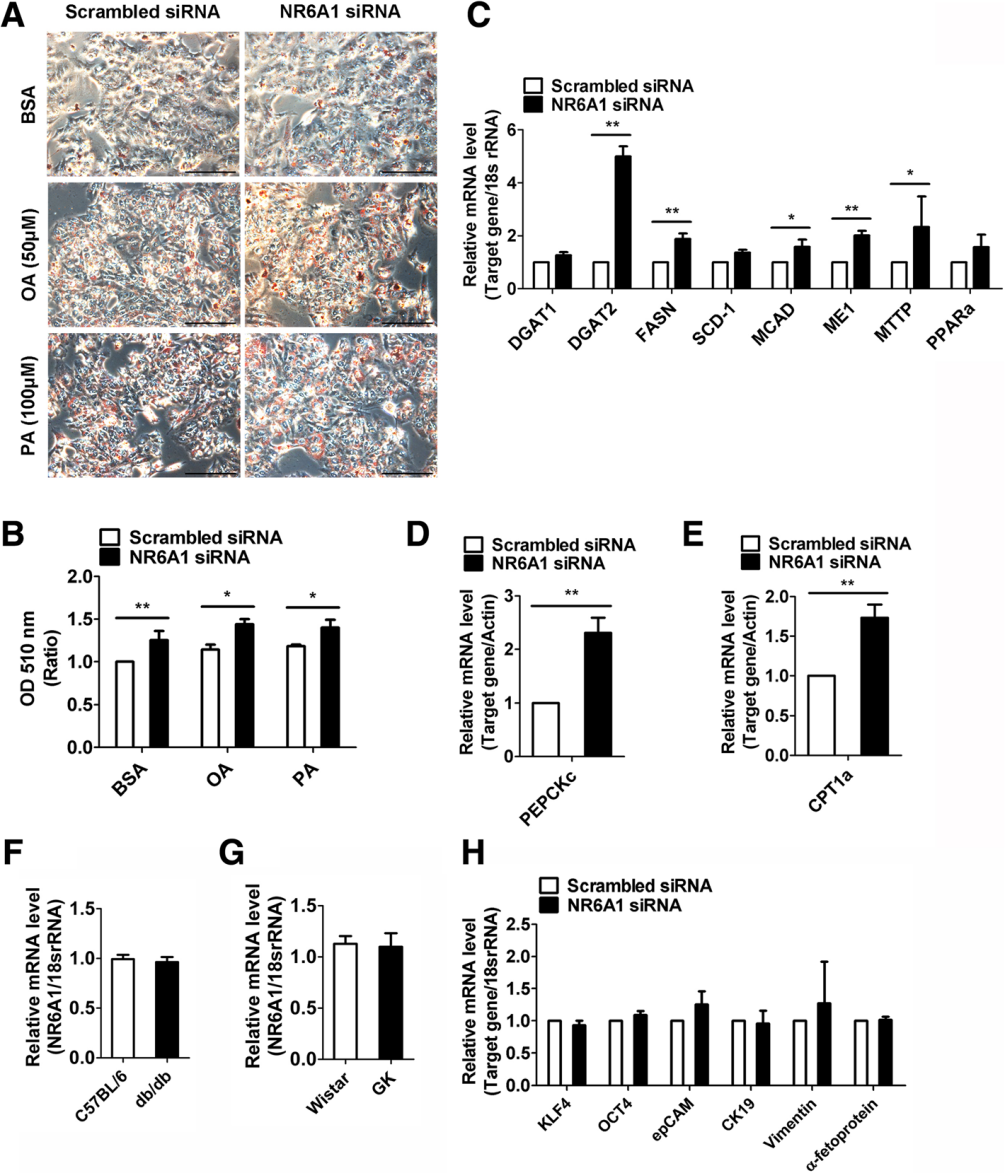
Effect of NR6A1 silencing on lipid content in HepG2 cells. HepG2 cells were transfected with scrambled or NR6A1 siRNA for 24 h and then incubated under normal growth medium or medium containing OA (50 μM) or PA (100 μM) for another 24 h. (**a**) Cells were fixed and stained by Oil Red O and then observed under fluorescence microscopy. Representative images were shown. Bar = 100 μm. (**b**) Quantification by extracting Oil Red O stained lipid droplets with 100% isopropanol and OD (510 nm) was measured. *n* = 3. **P < 0.01., *P < 0.05. HepG2 cells were transfected with scrambled or NR6A1 siRNA for 48 h. The mRNA levels of lipogenic genes (**c**), PEPCKc (**d**) and CPT1a (**e**) were determined by using qPCR method. Data are obtained from 3 independent experiments. **P < 0.01., *P < 0.05. (**f** & **g**)The animals were starved overnight for 16 h. After that, NR6A1 expression in liver was quantified by using qPCR method. (**f**) C57BL/6 vs. *db/db* mice (*n* = 4). (**d**) Wistar vs. GK rats (n = 4). (**h**) The expressions of cancer stem cell markers in control and NR6A1 silenced cells. *n* = 3

We next investigated the role of NR6A1 in lipid metabolism in HepG2 cells. The cells were transfected with NR6A1 siRNA for 48 h and then subjected to lipid content measurement with oil red O staining. The results showed that suppression of NR6A1 expression resulted in an increase in lipid content in HepG2 cells in all the conditions including normal culture medium or medium supplemented with PA or OA (Fig. 2A, B). We next examined the lipogenic gene expressions by using qPCR and observed that NR6A1 silencing promoted expressions of diglyceride acyltransferase-2 (DGAT2), fatty acid synthase (FASN), medium-chain acyl-coenzyme A dehydrogenase (MCAD), malic enzyme 1 (ME1) and microsomal triglyceride transfer protein (MTTP) (Fig. 2C). At the same time, we observed the expression of phosphoenolpyruvate carboxykinase (PEPCK), a rate limiting enzyme of gluconeogenesis in the liver, was also increased in NR6A1 silenced cells (Fig. 2D). Carnitine palmitoyltransferase 1A (CPT1a), which was upregulated in cancer cells, was also increased by NR6A1 knockdown (Fig. 2E). Given its potential role in insulin signaling, we examined the expression of NR6A1 in the livers of diabetes-resistant *db/db* mice and Goto-Kakizaki (GK) rats. Unexpectedly, NR6A1 levels did not show too much difference between livers of control and diabetes-resistant animals (Fig. 2F, G). Considering that NR6A1 can induce the repression of pluripotent genes, we checked the effect of NR6A1 on expressions of cancer stem cell markers in HepG2 cells and did not observe significant changes in cancer stem cell markers (Fig. 2H). These results demonstrated that loss of NR6A1 increases natural lipid level and lipid metabolic gene expression in HepG2 cells.

### NR6A1 regulates lipid metabolism in an mTORC1-dependent manner

**Fig. 3:**
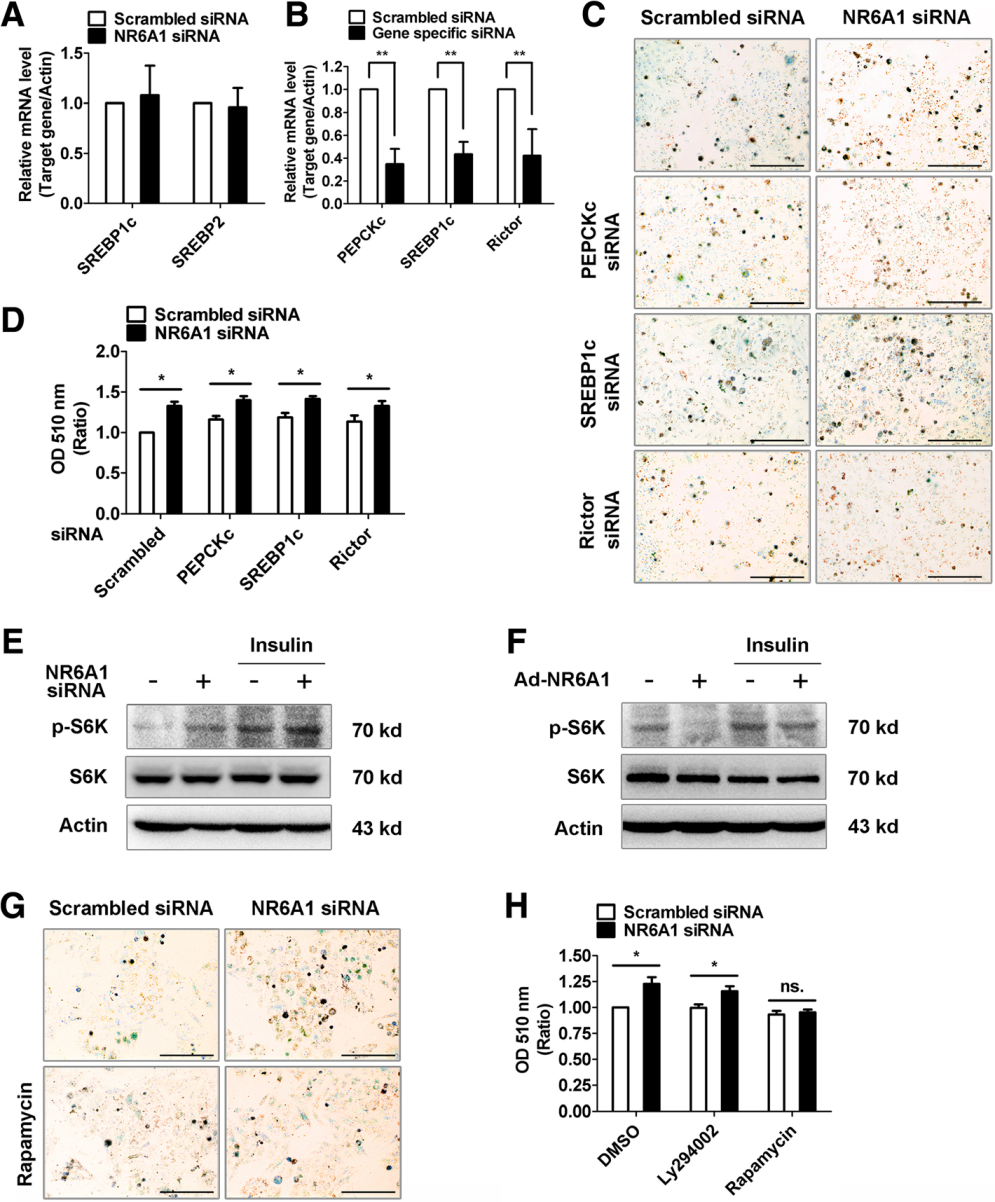
Regulation of lipid accumulation by NR6A1 is mTORC1 dependent. (**a**) HepG2 cells were transfected with control or NR6A1 siRNA fragments for 48 h, SREBP1c and SREBP2 transcripts were analyzed by using qPCR assay. *n* = 3. (**b**) HepG2 cells were transfected with scrambled, PEPCKc, SREBP1c or Rictor siRNA fragments for 48 h. The gene silencing was confirmed by using qPCR assay. *n* = 3. **P < 0.01. (**c**) Representative images of Oil Red O staining in HepG2 cells which transfected with NR6A1 siRNA alone or co-transfected with PEPCKc, SREBP1c or Rictor siRNA. Bar = 100 μm. *n* = 3. (**d**) Quantification by extracting Oil Red O stained lipid droplets with 100% isopropanol. *n* = 3. *P < 0.05. The phosphorylation of S6K in NR6A1 silenced- (**e**) or overexpressed (**f**) HepG2 cells were analyzed by using western blotting. Representative blots were shown. *n* = 3. (**g**) The control or NR6A1 silenced cells were treated with rapamycin for 16 h. Oil Red O staining was performed to show lipid content. Representative images were shown. Bar = 100 μm. *n* = 3. (**h**) Oil Red O contents in HepG2 cells. *n* = 3. *P < 0.05. ns. no significant

Cellular lipogenesis is controlled by both transcription factor SREBP, mTORC1 and mTORC2, the latter of which are downstream components of insulin. Recent research also showed that PEPCK drives lipogenesis in cancer cells [19]. We next investigated whether these key molecules are involved in NR6A1-modulated lipogenesis. We showed that NR6A1 knockdown did not alter the mRNA levels of SREBP1c or SREBP2 (Fig. 3A). Depletion of either PEPCKc, SREBP1c or Rictor failed to reverse the lipid accumulation in NR6A1 knockdown HepG2 cells (Fig. 3B-D). Previous studies also reported that activation of mTORC1 leads to increased storage of cellular natural lipids [20], which promoted us to evaluate the role of mTORC1 in NR6A1-modulated lipogenesis. Our results showed that NR6A1 knockdown can increase whereas NR6A1 overexpression can decrease p70-S6K phosphorylation, the downstream of mTORC1 (Fig. 3E, F). These data indicate that mTORC1 activity was regulated by NR6A1 in HepG2 cells. Indeed, inhibition of mTORC1 with rapamycin reversed the lipid accumulation in NR6A1 knockdown cells (Fig. 3G, H). Interestingly, a PI3K inhibitor Ly294002 did not show any effect in the lipd accumulation in NR6A1 knockdown cells. These data indicate that mTORC1 but not mTORC2 is required for lipid accumulation in NR6A1 silenced HepG2 cells.

### NR6A1 regulates insulin signal transduction in HepG2 cells

**Fig. 4:**
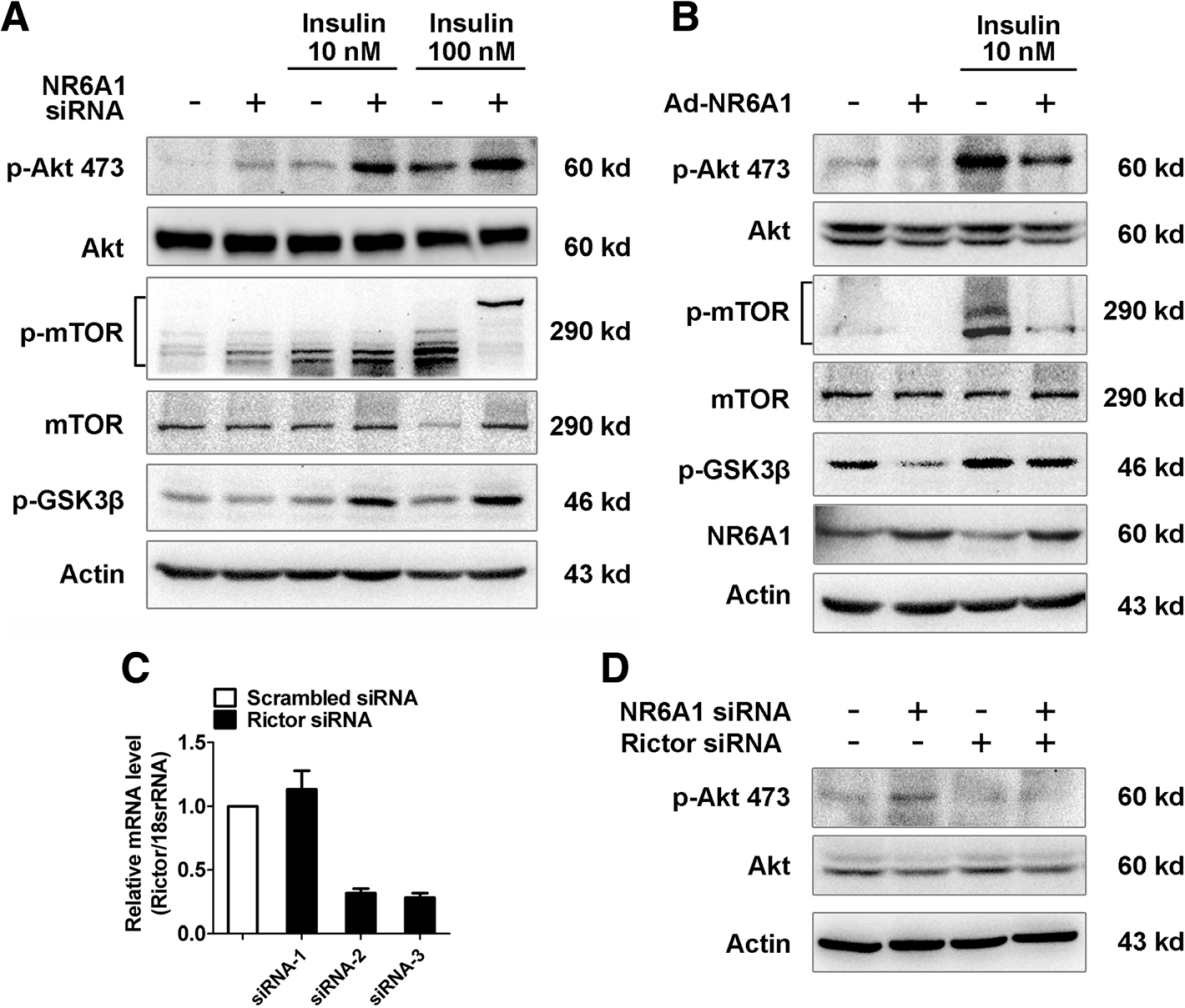
The effect of NR6A1 on insulin signaling in HepG2 cells. The control or NR6A1 silenced cells (**a**) and Ad-LacZ or Ad-NR6A1 infected cells (**b**) were cultured overnight in low serum (0.5% FBS) containing medium followed by treatment with insulin (10 nM or 100 nM) for 15 min. Cellular lysates were immunoblotted for AKT, phospho-AKT, mTOR, phospho-mTOR and phospho-GSK3β. Representative blots were shown. *n* = 3. (**c**) HepG2 cells were transfected with either scrambled or Rictor siRNA for 48 h. The mRNA expression of Rictor was analyzed by qPCR method. *n* = 3. (**d**) NR6A1 siRNA was cotransfected with either control siRNA or Rictor siRNA for 24 h, and starved overnight in low serum (0.5% FBS) medium before treated with insulin (10 nM) for 15 min. The expression of AKT and phospho-AKT were checked by western blotting. Representative blots were shown. *n* = 3

To investigate downstream insulin signaling activation, we studied the effect of NR6A1 on AKT, mTOR and GSK3β phosphorylation and observed that phosphorylation of AKT and mTOR showed greater levels in NR6A1 knockdown cells at both basal level and upon insulin stimulation. The phosphorylation of GSK3β, a downstream target of AKT, was increased in NR6A1 silenced cells (Fig. 4A). In contrast, cells overexpressing NR6A1 showed decreased activation of AKT, mTOR and GSK3β (Fig. 4B). To confirm the role of mTORC2 in NR6A1 silencing-induced AKT activation, we further knockdown Rictor, an essential component of mTORC2, and then determined the level of AKT phosphorylation. As shown in Fig. 4C & D, Rictor knockdown can inhibit NR6A1 silencing-induced phosphorylation of AKT, demonstrating a mTORC2-dependent AKT signaling in NR6A1 knockdown cells. Considering that insulin can stimulate lipogenesis, it is likely that NR6A1 knockdown can promote insulin receptor-dependent cellular anabolism through mTORC2 independent manner.

### INSR is required for NR6A1 silencing-induced lipid accumulation in HepG2 cells

**Fig. 5:**
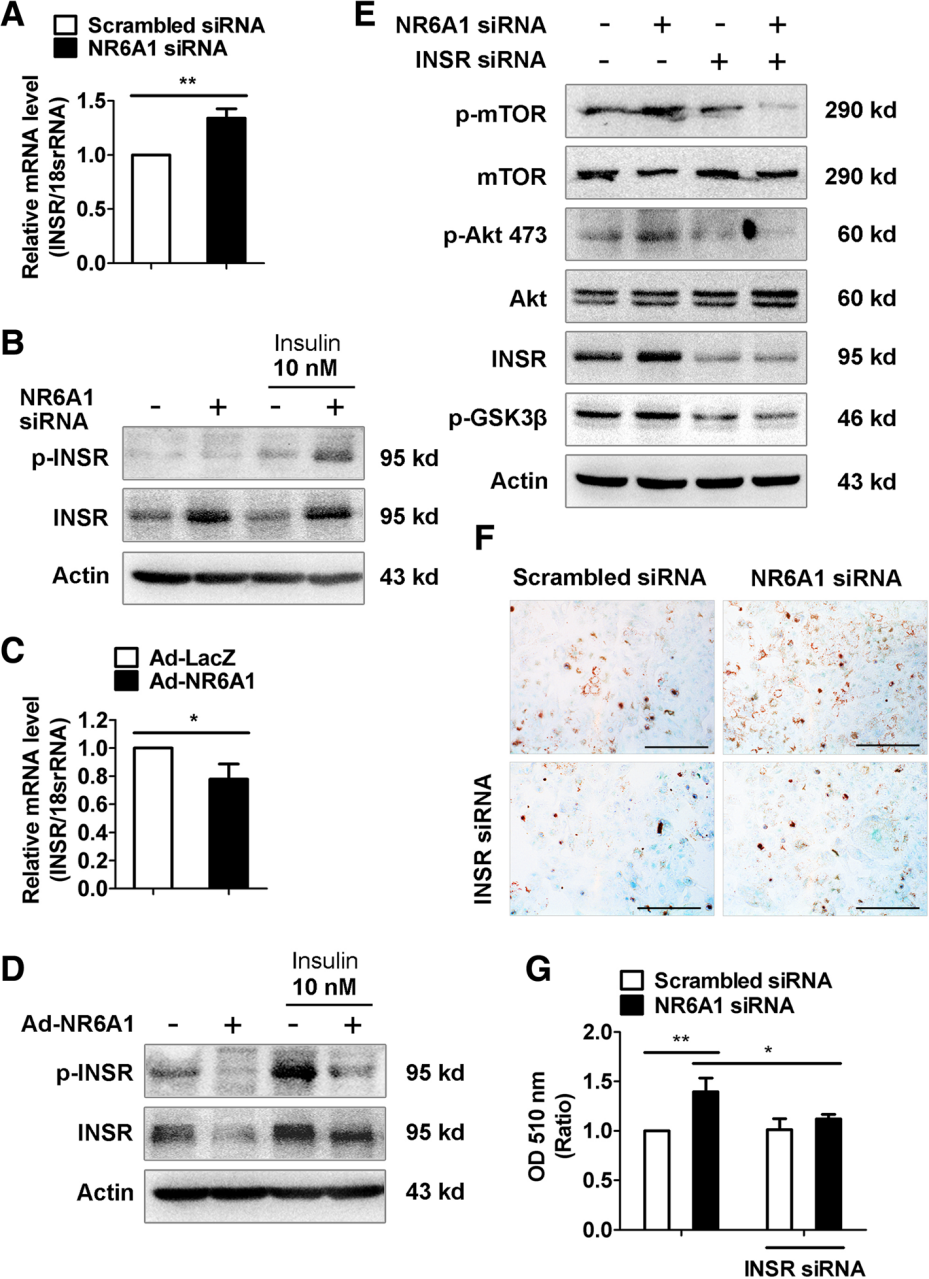
NR6A1 regulates INSR expression in HepG2 cells. (**a**) The mRNA expression of INSR in NR6A1 silenced HepG2 cells. *n* = 3. **P < 0.01. (**b**) HepG2 cells were transfected with scrambled or NR6A1 siRNA fragments for 24 h, and then starved in low serum (0.5% FBS) containing medium overnight before stimulated with insulin for another 15 min. Immunoblot analysis was performed to detect expressions of phosphorylated INSR and total INSR. Representative blots were shown. *n* = 3. (**c**) HepG 2 cells were infected with Ad-LacZ or Ad-NR6A1 for 48 h. The mRNA expression of INSR was analyzed by using qPCR method. *n* = 3. *P < 0.05. (**d**) Immunoblot analysis was performed to detect expressions of phosphorylated INSR and total INSR in Ad-NR6A1 infected cells. Representative blots were shown. *n* = 3. HepG2 cells were transfected with either scrambled or NR6A1 siRNA alone, or cotransfected with INSR siRNA for 48 h. (**e**) Total protein was collected for western blotting assay. Representative blots from 3 independent experiments were shown. (**f**) Oil Red O staining was performed to show the lipid accumulation. Representative images were shown. Bar = 100 μm. (**g**) Oil Red O contents in HepG2 cells. *n* = 3. **P < 0.01., *P < 0.05

We further examined whether NR6A1 increase mTORC downstream signaling by directly regulating transcription of mTORC components, but observed no significant changes in the expression of key mTORC components, such as TSC1, TSC2, Deptor, Misn1, mTOR, PRAS40, Raptor, PDK1 and Rictor, in NR6A1 silenced cells (data not shown). The mTORC1 and mTORC2/AKT primarily function as effectors of insulin signaling. The pathophysiology of lipid metabolism involves a complex network of INSR signaling pathways. We further elucidated whether the regulation of lipid accumulation by NR6A1 is rely on INSR. Our data showed that NR6A1 silencing increased whereas NR6A1 overexpression decreased the expression and phosphorylation of INSR in HepG2 cells at both basal levels and upon insulin stimulation (Fig. 5A-D). In addition, knockdown of INSR prevented NR6A1 silencing-induced phosphorylation of mTOR, AKT and GSK3β (Fig. 5E). Finally, knockdown of INSR reversed the lipid accumulation in NR6A1 knockdown cells (Fig. 5F & G). Collectively, our results suggest that NR6A1 modulates the expression and activation of INSR-dependent signaling to control lipogenesis in HepG2 cells.

### miR-205-5-p acts as a target of NR6A1 to increase lipid content in HepG2 cells

**Fig. 6:**
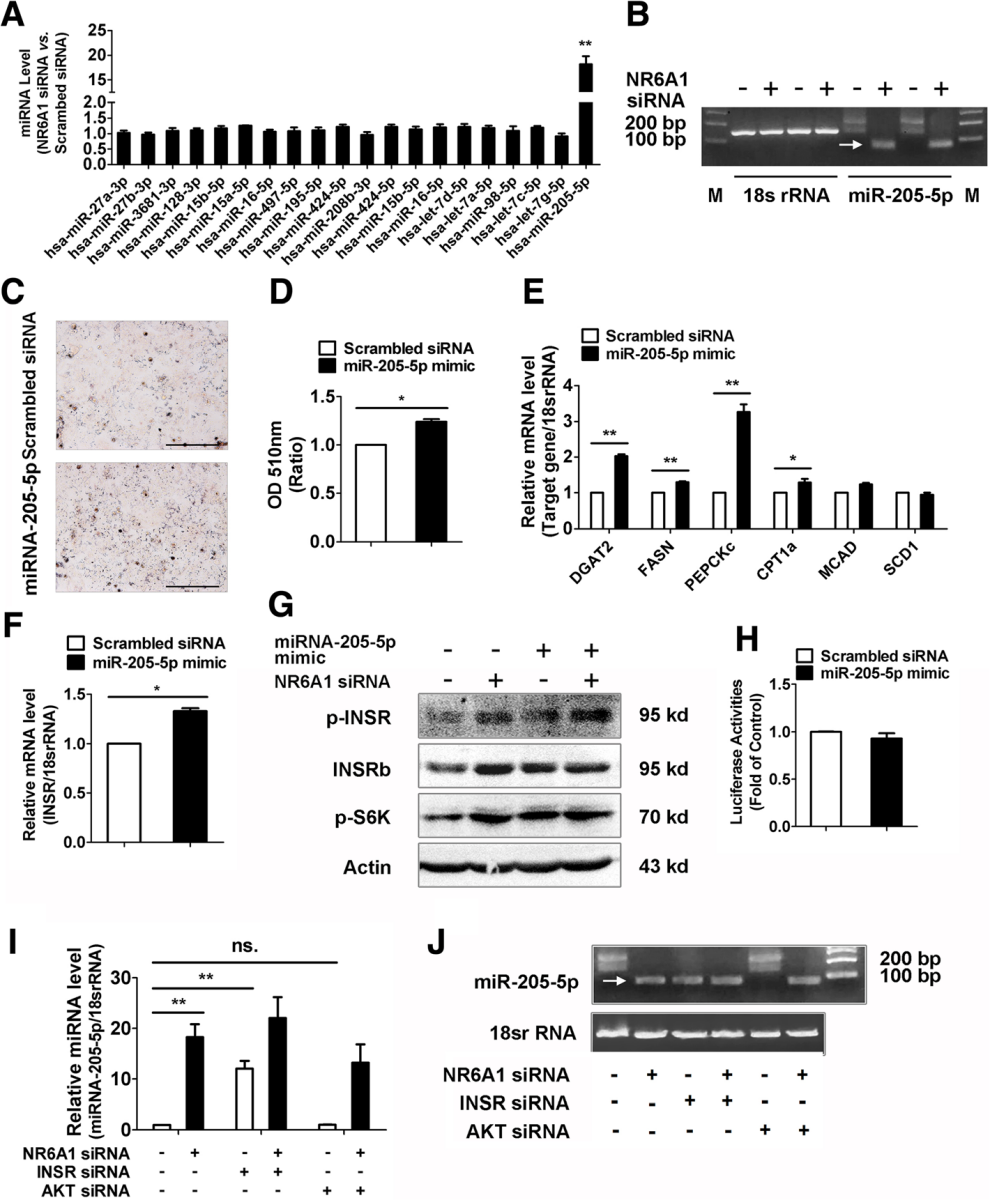
NR6A1 modulates miR-205-5p expression in HepG2 cells. (**a**) HepG 2 cells were transfected with scrambled or NR6A1 siRNA for 48 h. The expressions of a set of miRNA genes potential targeting INSR were checked by qPCR method. n = 3. **P < 0.01. (**b**) Gel electrophoresis of PCR product of miR-205-5p in control and NR6A1 silenced HepG2 cells. Representative image from 3 independent experiments was shown. (**c** & **d**) miRNA-205-5p mimic was transfected in HepG2 cells for 48 h. Oil Red O staining was performed to examine intracellular lipid content. Representative images were shown. Bar = 100 μm. *n* = 3. *P < 0.05. (**e** & **f**) DGAT2, FAS, PEPCKc, CPT1a, MCAD, SCD and INSR (**f**) mRNA levels in HepG2 cells in the presence or absence of 50 nM miR-205-5p mimics. *n* = 3. **P < 0.01., *P < 0.05. (**g**) Immunoblot analysis was performed to detect expressions of phosphorylated INSR, total INSR and phosphorylated p70-S6K in HepG2 cells which cotransfected with NR6A1 siRNA and miR-205-5p mimic. Representative images were shown. *n* = 3. (H) HepG2 cells were transfected with luciferase reporter plasmids containing the INSR 3’UTR combined with miR-205-5p mimic or miR-NC. *n* = 3. HepG2 cells were transfected with either scrambled or NR6A1 siRNA alone, or cotransfected with INSR or AKT siRNA for 48 h. (**i**) miR-205-5p expression was detected by qPCR method. (**j**) Representative gel electrophoresis of PCR products was shown. *n* = 3. **P < 0.01., *P < 0.05., ns. no significant

Given that NR6A1 silencing did not change the promoter activity and mRNA stability of INSR (data not shown), we then analyzed the expression of miRNAs targeting human INSR. The potential targets of miRNAs on human INSR were predicted by the online softwares (TargetScan and miRNABase). We checked the expression of twenty candidate miRNAs in NR6A1 knockdown cells and observed about eighteen folds increase in the expression level of miR-205-5p (Fig. 6A, B). Next, we utilized the miR-205-5p mimics to evaluate the effect of miR-205-5p on lipid content in HepG2 cells. The data showed that miR-205-5p mimic significantly induced cellular lipid accumulation (Fig. 6C, D). At the same time, miR-205-5p mimic increased the expressions of both lipogenic genes, such as DGAT2, FAS, PEPCKc and fatty acid oxidation gene CPT1a (Fig. 6E). These results indicate that miR-205-5-p can act as a NR6A1 target gene to regulate lipid metabolism in HepG2 cells.

Moreover, we observed that overexpression of miR-205-5p also increased the mRNA and protein expression of INSR (Fig. 6F, G). The miRNABase software showed a putative miR-205-5p binding site located at positions 525 to 531 bp of the 3′-UTR of INSR. To determine whether this predicated miRNA binding site is involved in miR-205-5p targeting of INSR in HepG2 cells, we transfected luciferase reporter construct containing INSR 3′-UTR into cells. The miR-205-5p mimic did not change the INSR 3′-UTR luciferase reporter activity (Fig. 6H), suggesting it is likely that miR-205-5p regulates expression of INSR through an indirect mechanism.

miR-205 was reported to regulate insulin downstream signaling through a homeostatic loop with Foxo. Next we sought to elucidate whether INSR can regulate miR-205-5p expression. Interestingly, siRNA-mediated knockdown of INSR but not its downstream effector AKT can significantly increase miR-205-5p expression and further enhanced miR-205-5p expression in NR6A1 knockdown HepG2 cells (Fig. 6I, J). These results suggested that INSR can regulate miR-205-5p expression in an AKT independent manner and there is a reciprocal regulation of miR-205-5p and INSR in HepG2 cells.

## Discussion

In this study, we identified a novel role NR6A1 in lipogenesis in hepatocellular carcinoma cells. Deregulation of lipid metabolism has been considered as an important metabolic hallmark of cancer cells. Numerous studies have indicated that cancer cells including hepatocellular carcinoma cells are closely tied to the enhanced lipid metabolism to meet their abnormal metabolic requirements. Activation of de novo lipogenesis in cancer cells is implicated in the production of membranes for rapid cell proliferation, and also for alterations on intracellular oncogenic signaling and endoplasmic reticulum homeostasis [21–23]. In addition, the increasing lipid accumulation in cancer cells plays important roles in activation of adaptive responses to harmful stimuli, such as free radicals and chemotherapeutics [24, 25]. On the contrary, inhibition of lipid synthesis decreases cancer cell proliferation and invasion and renders cancer cells susceptible to oxidative stress-induced cell death. It was noted that a number of nuclear factors, such as FXR, PPARs, RXR, PXR, HNF and NR4A1 [26–28], which play critical roles in the regulation of lipid metabolism, contribute to hepatocellular carcinoma development. Here, we reported that NR6A1 may also be a potential lipid metabolic regulator in HepG2 cells.

Our results demonstrated the regulation of NR6A1 on FAS and DGAT2 in HepG2 cells, both of which are key lipogenic enzymes and have been shown to drive caner development. It is well known that lipids are generated from uptake from extracellular environment, lipolysis, and de novo fatty acid synthesis in cancer cells. Increased expression of FAS, which catalyzes the last step in the de novo fatty acid biosynthetic pathway, has been observed in many types of cancers including hepatocellular, stomach, colorectal, lung, breast and thyroid cancers [29]. FAS is involved in the primary synthesis of 16-carbon fatty acid, from which other forms of lipids are also synthesized. It was shown that tumor-associated FAS promotes cancer cell proliferation and survival rather than functions as a regulator of anabolic energy-storage pathway [21]. FAS inhibition has been shown to induce apoptosis of cancer cells and cancer growth delay in vivo [30]. Another enzyme involving in fatty-acid and cholesterol biosynthesis, DGAT2, is mainly expressed in the liver and white adipose tissue and responsible for the last step of triglycerides (TG) synthesis. Hepatic DGAT2 overexpression was found to induce TG content accumulation in the liver. On the contrary, inhibition of DGAT1 and DGAT2 disrupts TG fatty acid composition and compromises in vivo cancer growth by increasing apoptosis and reducing proliferation of cancer cells [31].

In addition, glucose metabolism is known to link with de novo lipogenesis during intracellular TG formation in cancer cells. Likewise, ME1 converts malate to pyruvate and produces nicotinamide adenine dinucleotide phosphate, which is essential for fatty acid synthesis. Higher level of ME1 was shown to regulate glucose metabolism and protect cancer cell apoptosis [32]. PEPCK, a regulator of gluconeogenesis and tricarboxylic acid cycle flux, was recently found to increase cancer growth by promoting the utilization of glucose and glutamine toward anabolic metabolism and enhancing lipogenesis in cancer cells [19]. Although in our study, PEPCK knockdown cannot reverse NR6A1 silencing-induced lipid accumulation in HepG2 cells, it may promote cell growth by increasing glucose metabolism. On the other hands, lipid oxidation is an important component of cancer metabolism together with lipogenesis, and is associated with proliferation and prognosis in cancers. CPT1a controls the rate-limiting step of fatty acid oxidation by facilitating the entry of fatty acids into the mitochondria. It may promote fatty acid β-oxidation to generate excess ATP and induce the hepatocellular carcinoma cell aggressive phenotype. Blocking CPT1a results in cancer cell death and decreased resistance to radiation and chemotherapeutic agents of cancer cells [33, 34]. The expressions of lipid metabolism enzymes are controlled by transcriptional repressors or activators, including ChREBP, SP1, ARP-1/COUP-TF II, SREBP-1, liver X receptors and et al. But little is known about DGAT2 transcriptional control in liver and the regulation of other enzyme expressions still remain to be elucidated. We herein showed that NR6A1 can regulate both catabolic and anabolic activity in HepG2 cells by regulating lipid metabolism enzyme expression.

Insulin is a major anabolic hormone and also a growth factor. It acts directly by binding to INSR to activate mTORC2/AKT and further stimulate lipogenesis through mTORC1-dependent and independent pathways in hepatocytes [35]. We observed that NR6A1 silencing increased INSR downstream AKT phosphorylation through mTORC2. However, knockdown of Rictor, the rapamycin-insensitive companion of mTOR, cannot inhibit NR6A1 silencing-induced lipid accumulation in HepG2 cells, suggesting the presence of AKT-independent signaling steps necessary for the lipid metabolism in NR6A1-silenced HepG2 cells. Additionally, both rapamycin treatment and INSR knockdown can reduce lipid accumulation in NR6A1 silenced HepG2 cells. These data indicated that NR6A1 regulates lipid metabolism in HepG2 cells by mTORC1 dependent manner, which also rely on intracellular insulin signaling pathway other than AKT.

The experiments have shown that INSR expression can be directly targeted by several miRNAs, such as miR-424-5p, miR-15b, miR-1271, miR-195, miR-497 and miR-96 in HepG2 or other type cells [36–38]. In addition, gene expression may also be regulated indirectly by miRNAs. miR-205-5p has been shown to be involved in insulin signaling but not alter hepatic fat content in primary hepatocytes. It regulates insulin signaling by inhibiting Foxo1 expression and increasing AKT phosphorylation in liver [39]. However, endogenous miR-205 was also found to regulate lipogenesis by inhibiting glycogen synthase kinase 3β in adipose tissues [40]. Another study uncovered that miR-205 deregulates lipid metabolism through targeting acyl-CoA synthetase long-chain family member 1 in hepatoma cells [41]. Here, we reported that miR-205-5p was a NR6A1 target gene, and can induce hepatic lipogenesis by up-regulating lipogenic and INSR gene expression in HepG2 cells. Considering that there is not any DR0 sequence in miR-205-5p gene, we proposed that NR6A1 indirectly regulates miR-205-5p expression. We also observed no difference in NR6A1 mRNA expression between control and hyperinsulinemic *db/db* mice or diabetic rats, indicating that NR6A1 is more likely is involved in insulin signaling in HepG2 cells. Foxo can regulate miR-205 expression in hepatic cells through an indirect mechanism [39]. We further described a feedback loop between INSR and miR-205-5p in HepG2 cells.

## Conclusions

The present study demonstrates that NR6A1 regulates lipid metabolism through a mTORC1 dependent pathway. We confirm the role of NR6A1 as an important repressor of lipogenesis and INSR gene expression in HepG2 cells. In addition, we show that there is a reciprocal regulation loop between nuclear receptor, membrane receptor and miRNA, both of which controls lipid metabolism in HepG2 cells.

## Additional file

Additional file 1: **Table S1.** The sequences of primers and siRNAs. (XLS 29 kb)

## Data Availability

The data generated or analyzed during this study are included in this article, or if absent are available from the corresponding author upon reasonable request.

## Abbreviations

CPT1a: Carnitine palmitoyltransferase 1A
DGAT2: Diglyceride acyltransferase-2
FAS: Fatty acid synthase
GSK3β: Glycogen synthase kinase 3 β
INSR: Insulin receptor
ME1: Malic enzyme 1
miRNA: MicroRNA
mTORC: Mammalian target of rapamycin complex
MTTP: Microsomal triglyceride transfer protein
NR6A1: Nuclear receptor sub-family 6, group A, member 1
PEPCK: Phosphoenolpyruvate carboxykinase
qRT-PCR: Quantitative real-time polymerase chain reaction
S6K: S6 kinase
UTR: Untranslated region
